# Excretory/Secretory Products From *Trichinella spiralis* Adult Worms Attenuated DSS-Induced Colitis in Mice by Driving PD-1-Mediated M2 Macrophage Polarization

**DOI:** 10.3389/fimmu.2020.563784

**Published:** 2020-10-02

**Authors:** Zixia Wang, Chunyue Hao, Qinghui Zhuang, Bin Zhan, Ximeng Sun, Jingjing Huang, Yuli Cheng, Xinping Zhu

**Affiliations:** ^1^Department of Medical Microbiology and Parasitology, School of Basic Medical Sciences, Capital Medical University, Beijing, China; ^2^Department of Pediatrics, National School of Tropical Medicine, Baylor College of Medicine, Houston, TX, United States; ^3^Department of Clinical Laboratory Medicine, Guangdong Provincial People’s Hospital, Guangdong Academy of Medical Sciences, Guangzhou, China

**Keywords:** inflammatory bowel disease, *Trichinella spiralis*, macrophages, programmed death 1, excretory/secretory products

## Abstract

Helminth-modulated macrophages contribute to attenuating inflammation in inflammatory bowel diseases. The programmed death 1 (PD-1) plays an important role in macrophage polarization and is essential in the maintenance of immune system homeostasis. Here, we investigate the role of PD-1-mediated polarization of M2 macrophages and the protective effects of excretory/secretory products from *Trichinella spiralis* adult worms (AES) on DSS-induced colitis in mice. Colitis in mice was induced by oral administration of dextran sodium sulfate (DSS) daily. Mice with DSS-induced colitis were treated with *T. spiralis* AES intraperitoneally, and pathological manifestations were evaluated. Macrophages in mice were depleted with liposomal clodronate. Markers for M1-type (iNOS, TNF-α) and M2-type (CD206, Arg-1) macrophages were detected by qRT-PCR and flow cytometry. Macrophage expression of PD-1 was quantified by flow cytometry; RAW 264.7 cells and peritoneal macrophages were used for *in vitro* tests, and PD-1 gene knockout mice were used for *in vivo* investigation of the role of PD-1 in AES-induced M2 macrophage polarization. Macrophage depletion was found to reduce DSS-induced colitis in mice. Treatment with *T. spiralis* AES significantly increased macrophage expression of CD206 and Arg-1 and simultaneously attenuated colitis severity. We found *T. spiralis* AES to enhance M2 macrophage polarization; these findings were confirmed studying *in vitro* cultures of RAW264.7 cells and peritoneal macrophages from mice. Further experimentation revealed that AES upregulated PD-1 expression, primarily on M2 macrophages expressing CD206. The AES-induced M2 polarization was found to be decreased in PD-1 deficient macrophages, and the therapeutic effects of AES on colitis was reduced in PD-1 knockout mice. In conclusion, the protective effects of *T. spiralis* AES on DSS-induced colitis were found to associate with PD-1 upregulation and M2 macrophage polarization. Thus, PD-1-mediated M2 macrophage polarization is a key mechanism of helminth-induced modulation of the host immune system.

## Introduction

Epidemiological studies are increasingly reporting an inverse association between the prevalence of autoimmune diseases and helminth infections. There has been growing interest in exploiting the immunoregulatory capabilities of helminths to develop novel therapies for treatment of autoimmune inflammatory diseases and allergic diseases (1). Ulcerative colitis and Crohn’s disease are two well-established forms of inflammatory bowel diseases (IBD) characterized as complex, immune-mediated disorders (2). Prior research has described the protective effects of helminth infections and specifically helminth-derived proteins on various IBD in animal models as well as placebo-controlled human clinical trials (3, 4), suggesting the potential application of helminth infection or helminth-derived products in IBD therapy. Mechanistic studies to date have mainly focused on the roles of type 2 immunity mediated by Th2 and regulatory T cells (4–6). However, some reports have implicated that the macrophage populations are involved in helminth-generated protection against intestinal pathology (7–9).

Macrophages are a versatile cell population that plays vital roles in clearing bacteria from local tissues, translating alert signals to other immune cells, secreting cytokines to establish local homeostatic immune cell networks, and participating in T cell restimulation and maintenance within the lamina propia (10). Activated macrophages are functionally divided into two main categories: the M1 type (the classical-activated macrophage) and the M2 type (the alternatively activated macrophage) (11). Although M1-type macrophages release proinflammatory cytokines and enhance inflammatory responses, they also cause injury to host tissue (12). On the contrary, M2-type macrophages release anti-inflammatory cytokines and help maintain tissue immune homeostasis, thus avoiding overactive inflammation (13, 14). In the fight against foreign pathogens, the shifting balance between proinflammatory M1 and wound-healing M2 macrophages over time is essential for proper resolution of inflammation (15). In addition, M2-type macrophages have been reported to attenuate experimentally induced inflammation in the gut (16). Passive transfer of *in vitro* derived alternatively activated macrophages conferred significantly reduced colonic inflammation (16). Helminth-modulated macrophages have been explored as promising therapeutic for inflammatory disease (8, 16–18).

The programmed cell death-1 (PD-1) is a member of the CD28 superfamily that delivers negative signals upon interaction with its two ligands, PD-L1 and PD-L2. Expression of PD-1 on T cells, B cells, macrophages, and dendritic cells (DCs) is inducible (19). The PD-1 pathway is vital for physiologic regulation of immune responses to avoid injurious overactive inflammation (20, 21). In addition, PD-1 serves as an important immune checkpoint to keep immune balance and prevent autoimmunity (21–23). Although many studies have shown the importance of PD-1 in modulating the T cell function, some evidence has emerged that PD-1 plays a distinct role in the regulation of macrophage polarization (24, 25). Expression of PD-1 was reported to promote macrophage polarization toward M2 phenotype and PD-1 deficiency enhanced M1 polarization (26, 27).

In recent years, many studies have indicated that helminths induce PD-1 pathway to modulate the host immune system to minimize excessive inflammation against invaded parasites facilitating worms’ survival and promoting the chronicity of helminth infection (28). *Trichinella spiralis*, a tissue-dwelling intestinal nematode, is known to secrete molecules that modulate the host immune system (29). Infection with this nematode, or treatment with *Trichinella*-derived proteins, has been extensively investigated for the treatment of many hypersensitivity disorders (7, 30, 31). Our previous study demonstrated that excretory/secretory products from *T. spiralis* adult worms (*Ts*-AES) ameliorated DSS-induced colitis in mice *via* inhibiting proinflammatory cytokines (IFN-γ, IL-6, IL-17) (5). It has been reported that adoptive transfer of macrophages obtained from helminth-infected mice and helminth ES protein-activated macrophages reduced allergic asthma and DSS-induced colitis in mice (16). However, the signal pathway(s) and mechanism involved in the M2 macrophage polarization induced by *T. spiralis* AES are still unknown. Here, we characterize the role of *T. spiralis* AES in the M2 macrophage polarization associated with the therapeutic efficacy of DSS-induced colitis in mice and the involvement of PD-1 in the activation of M2 macrophages. This study highlights the importance of PD-1 as a checkpoint for AES-induced M2 macrophage polarization associated with the protective effects of AES on DSS-induced colitis. Our findings provide new insights into the mechanisms of helminth immunomodulation of the host immune system and the potential therapeutic effect of nematode-derived proteins on inflammatory bowel diseases.

## Materials and Methods

### Animals

Wild-type (WT) and PD-1 knockout mice (KO mice) of the C57BL/6 strain were purchased from Jackson Laboratory (stock no. 021157, USA). Female ICR mice aged 6–8 weeks and rats were purchased from the Capital Medical University Laboratory Animal Services Center (Beijing, China). All animals were kept in a pathogen-free environment.

### Preparation of Excretory/Secretory (ES) Products From Adults

*T. spiralis* (strain ISS 533) was maintained in female ICR mice. The muscle larvae were recovered from infected mice by digestion of the carcasses in artificial gastric juice (32). The recovered muscle larvae were used to infect rats orally with 14,000 larvae each. Six days after infection, each rat was euthanized, and the adult *T. spiralis* worms were collected from intestine. The collected adult worms were washed several times with sterile saline (0.9% NaCl) and cultured in RPMI-1640 (Thermo Fisher, Carlsbad, USA) supplemented with 200 U penicillin/ml and 200 μg streptomycin/ml (Caisson labs, Logan, USA) at 3000 worms/ml at 37°C, 5% CO_2_, for 48 h. Cultured medium containing AES products was collected and concentrated buffer-exchanged to PBS using a centrifugal filter (Millipore Amicon Ultra-15, NMWL: 3000, USA), and buffer-exchanged to PBS (32). Protein concentration in AES was determined using the BCA protein assay (Merck, Darmstadt, Germany).

### Development of a DSS-Induced Colitis Mouse Model

**Figure 1:**
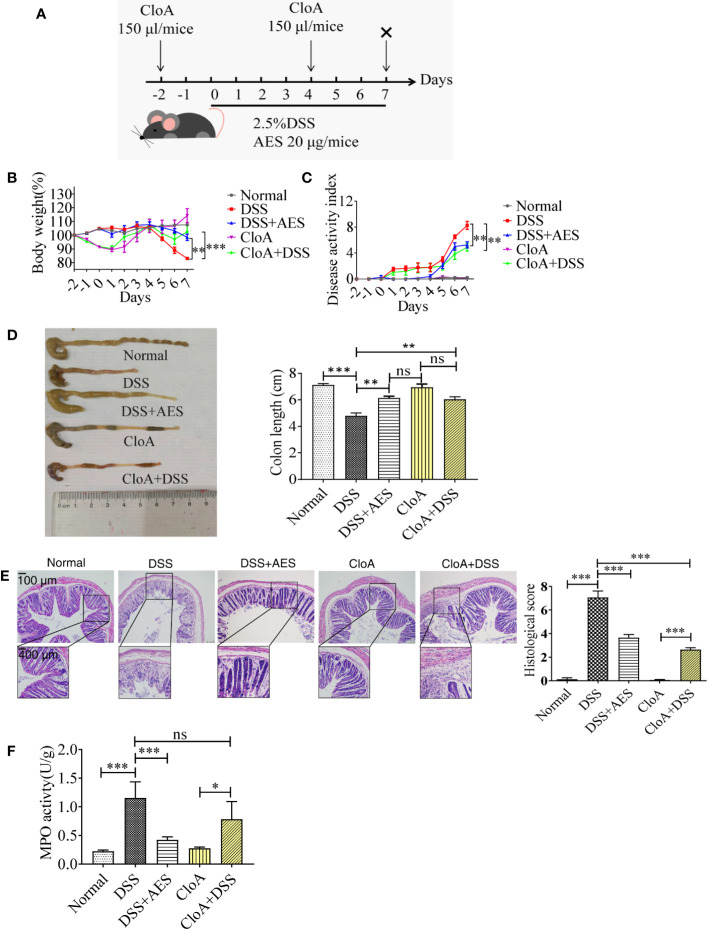
*T. spiralis* AES alleviated DSS-induced colitis in mice. Mice were divided into 5 groups with 6 mice each, treated with DSS, DSS+AES, CloA, and CloA+DSS, respectively. Another group of 6 mice were given PBS as normal control. **(A)** The detailed study regimen including time points for administration of DSS, *T. spiralis* AES, and CloA. **(B)** Percentage of mouse weight loss after treatment (n=6). **(C)** Clinical disease activity index (DAI) was assessed (n=6). **(D)** Representative colonic length from each group (left) and average reduction in colonic length (right) (n=6). **(E)** H&E staining of representative colon samples from each group (left) and the changes in histological score in each group is shown on the right (n=6). **(F)** Myeloperoxidase activity in mouse colons of each group (n=6). Experiments were repeated in triplicate. Data are expressed as means ± SEM. “**×**” indicates sacrificing of mice. *indicates significant differences between experimental groups and controls. ^*^P < 0.05, ^**^P < 0.01, ^***^P < 0.001; ns = not significant.

To develop a colitis mouse model, male C57BL/6 mice were exposed to 2.5% dextran sodium sulfate polymers (DSS, 36,000–50,000 MW, MP Biomedicals, Solon, USA) in drinking water for 7 days. Meanwhile, each mouse was injected intraperitoneally with 20 μg AES in a total volume of 100 µl every day until day 7, when the mice were sacrificed (**Figure 1A**). The same volume of PBS was administered to control mice. Animal body weights, stool consistency (diarrhea), and presence of rectal bleeding were monitored daily. All mice were sacrificed on day 7. Colons were collected, and their length was measured from cecum to rectum. The disease activity index (DAI) was scored based on the following parameters: stool consistency (0–4), presence of fecal blood (0–4), and percentage of body weight loss (0–4) as previously described (5).

To evaluate the involvement of macrophages in DSS-induced colitis, liposomal clodronate was used to selectively and efficiently deplete macrophages from treated mice (33). In this experiment, each mouse was injected intraperitoneally with 1 mg of clophosome-A (CloA), an anionic liposomal clodronate (FormuMax, Sunnyvale, USA), in a volume of 150 µl 2 days prior to (day -2) and 4 days after (day 4) DSS exposure.

### Measurement of Myeloperoxidase Activity

To measure the activity of myeloperoxidase (MPO), an enzyme present nearly exclusively in neutrophils, an MPO assay kit (Nanjing Jiancheng Bio-engineering Institute, China) was used according to the manufacturer’s instructions. Briefly, 50 mg of colon tissue was homogenized by ultrasonication in 500 μl of 0.9% NaCl. Then, MPO activity was measured in 25 µl of colon tissue homogenate as U/g protein.

### Histological Analysis

Colonic tissue proximal to the rectum was collected from each mouse and fixed in 4% paraformaldehyde fixation solution. Sections were stained with hematoxylin and eosin. The histological damage score was calculated using two parameters: epithelial damage (0–4) and inflammatory cell infiltration (0–4) as described previously (5).

### Peritoneal Macrophages

Peritoneal macrophages were isolated from C57BL/6 male mice (WT) and PD-1 KO mice by washing the peritoneal cavity twice with 5 ml Hank’s buffered salt solution (HBSS without calcium and magnesium, Gibco, Grand Island, USA). Harvested peritoneal cells were cultured in 6-well tissue culture plates with 2×10^6^ cells per well in RPMI-1640 medium supplemented with 0.5% inactivated FBS, 100 U penicillin/ml and 100 μg streptomycin/ml; cells were allowed to adhere for 4 h, and nonadherent cells were removed. Adherent macrophages were collected directly for flow cytometry or treated with AES (4 μg/mL) for 2 h and then with either IL-4 (20 ng/ml) or LPS (100 ng/ml) (PeproTech, Rocky Hill, NJ, USA) and 10% inactivated FBS for an additional 6 h. Cells were finally collected for analysis.

The RAW 264.7 murine macrophages were obtained from the Chinese National Infrastructure of Cell Line Resource (http://cellresource.cn/contact.aspx, China) and cultured and analyzed under conditions identical to those of peritoneal macrophage culturing.

### RNA Extraction and qRT-PCR

Total RNA was extracted from macrophages by TRIzol reagent and quantified by absorbance at 260 nm. A Fast King RT Kit (TianGen, Beijing, China) was used to reverse-transcribe mRNA to cDNA. All qRT-PCR reactions were performed in triplicate using the TransStart Top Green qPCR SuperMix kit (TransGen, Beijing, China) and QuantStudio-5 (Applied Biosystems, Thermo Fisher Scientific, USA). Primer sequences used for PCR (Invitrogen, Shangahi, China) were as follows: mouse GAPDH, ACCCAGAAGACTGTGGATGG (forward) and CACATTGGGGGTAGGAACAC (reverse); mouse *Cd206*, TCTTTGCCTTTCCCAGTCTCC (forward) and TGACACCCAGCGGAATTTC (reverse); mouse *Arg1*, AGACAGCAGAGGAGGTGAAGAGTAC (forward) and GGTAGTCAGTCCCTGGCTTATGGT (reverse); mouse *inos*, CCCTTCAATGGTTGGTACATGG (forward) and ACATTGATCTCCGTGACAGCC (reverse); mouse *Tnfα*, TCTTCTCATTCCTGCTTGTGG (forward) and GGTCTGGGCCATAGAACTGA (reverse). Fold induction of target gene expression was calculated using the comparative method by normalization to the internal control GAPDH.

### Flow Cytometry

The following reagent and antibodies were purchased from Thermo Fisher Scientific (Carlsbad, USA) and used in flow cytometry analysis: fixable viability dye (FVD-eFluor 506), antimouse F4/80 (FITC), antimouse CD11b (PE-Cy7), antimouse CD206 (APC), antimouse iNOS (PE), anti-CD279 (PD-1) (PerCP-eFluor 710), and CD16/CD32 monoclonal antibody.

For M1/M2 macrophage phenotype analysis, 2×10^6^ cells were stained with anti-F4/80-FITC, anti-CD11b-PE-Cy7, and FVD-eFluor 506) for 30 min at 4°C after mouse Fc was blocked (CD16/CD32) for 20 min. After fixation and permeabilization, intracellular staining was carried out with anti-CD206-APC and anti-iNOS-PE. Cells that were FVD^-^ were identified as living; F4/80^+^ and CD11b^+^ cells were identified as macrophages; F4/80^+^ CD11b^+^ CD206^+^ cells were identified as M2-type macrophages; and F4/80^+^ CD11b^+^iNOS^+^ cells were identified as M1-type macrophages.

All cells were subsequently detected using a BD LSRFortessa flow cytometer (BD Biosciences, Heidelberg, Germany), and data were analyzed with FlowJo software (BD Biosciences).

### Statistical Analysis

GraphPad Prism version 7 software (San Diego, CA, USA) was used to analyze statistical difference between groups. Results are presented as means ± SEM. Comparisons between groups were performed using one-way ANOVA or unpaired two-tailed Student’s *t*-tests; *P* < 0.05 was considered statistically significant.

## Results

### *T. spiralis* AES Alleviated DSS-Induced Colitis

Mice were administered with DSS daily to induce colitis. A group of mice was treated with 20 µg of AES by intraperitoneal injection simultaneously (**Figure 1A**). Seven days after treatment, mice administered with DSS manifested with typical pathological changes of colitis compared to the normal control mice, represented by more serious disease activity, weight loss, bleeding diarrhea, and colon shortness (**Figures 1B–D**). Histological observation also showed that DSS-exposed mice demonstrated serious epithelia disruption, disappearance of intestinal crypts and goblet cells, marked mucosal hypertrophy, and edema and inflammatory cell infiltration in colon tissue (**Figure 1E**). MPO activity was also significantly increased in mice treated with DSS (**Figure 1F**). All results indicate that DSS induced serious colon inflammation and pathology. Pathological severity of colitis, however, was significantly reduced in the group of mice treated with AES as characterized by reduced body weight loss, alleviated clinical manifestations (DAI), decreased histological score and MPO activity as compared to mice without AES treatment (**Figures 1B–F**). Macrophage depletion by CloA mitigated all aforementioned clinical signs of colitis in DSS-exposed mice, including reduced macroscopic and microscopic DSS-induced pathology (**Figures 1B–F**). Treatment with CloA did not change MPO activity in the intestines of mice with colitis possibly because MPO activity mainly reflects the function of neutrophil cells, not macrophages (**Figure 1F**) (34). Our findings suggest that macrophages are involved in the pathogenesis of DSS-induced colitis, consistent with Weisser’s observation that showed M1 macrophages contributed to DSS-induced colitis (35).

### *T. spiralis* AES Stimulated M2 Macrophage Polarization in Mice With DSS-Induced Colitis

**Figure 2:**
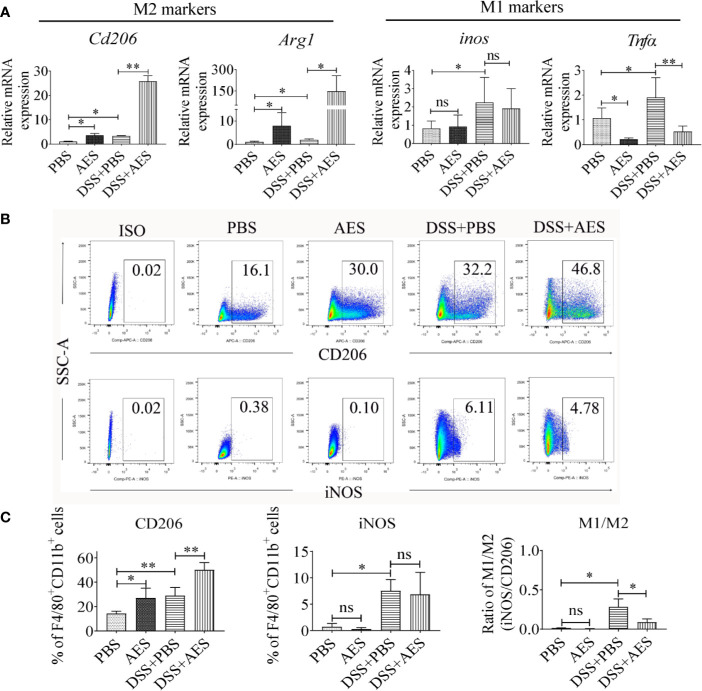
Treatment with AES stimulated M2 macrophage polarization in mice with DSS-induced colitis. Each group of mice (n=6) was treated with DSS, AES, DSS+AES, and PBS, respectively. **(A)** The mRNA expression of M2 (*Cd206, Arg1*) and M1 (*inos, Tnfα*) associated genes in peritoneal macrophages collected from each treated group were measured by qRT-PCR (n=6). The relative expression level was compared with PBS control group as a baseline. **(B)** Peritoneal macrophages collected from each mouse group were analyzed by flow cytometry. The representative side scatter area (SSC-A) plots are shown for CD206 (M2) and iNOS (M1) labeling (n=6 per group). **(C)** The percentage of CD206 (M2) and iNOS (M1) expressed macrophages (F4/80^+^, CD11b^+^) in each group (n=6) (left, middle) and their M1/M2 ratio in each mouse group (n=6) (right). Experiments were repeated in triplicate. Data are expressed as means ± SEM; ^*^P < 0.05, ^**^P < 0.01; ns = not significant.

The qRT-PCR analysis of peritoneal macrophages collected from treated mice revealed that DSS exposure led to a significant increase in the expression of M1 genes (*inos, Tnfα*); however, treatment with AES significantly upregulated M2 gene expression (*Cd206, Arg1)* and downregulated M1 gene *Tnfα* expression in macrophages from DSS-induced mice with colitis as compared to mice without AES treatment (**Figure 2A**). However, there was no significant change in *inos* mRNA expression between mice with and without AES treatment. Results of flow cytometry analysis were consistent with qRT-PCR results showing that macrophages from mice with DSS-induced colitis expressed higher levels of both M1 (F4/80^+^, CD11b^+^ and iNOS) and M2 (F4/80^+^, CD11b^+^ and CD206) macrophage markers than those in control mice. Treatment with AES significantly induced M2 marker (CD206) expression on peritoneal macrophages collected from both DSS-treated or nontreated mice, but induction was more significant in DSS-treated mice. No significant change in M1 marker (iNOS) expression was noted (**Figures 2B, C**) although the M1/M2 ratio remained significantly decreased in mice treated with AES as compared to untreated mice (**Figures 2C**). These data suggest that AES treatment enhanced macrophage polarization toward the anti-inflammatory and regulatory M2 type, thus alleviating colitis severity.

### *T. spiralis* AES Enhanced IL-4-Induced M2 Macrophage Polarization In Vitro

**Figure 3:**
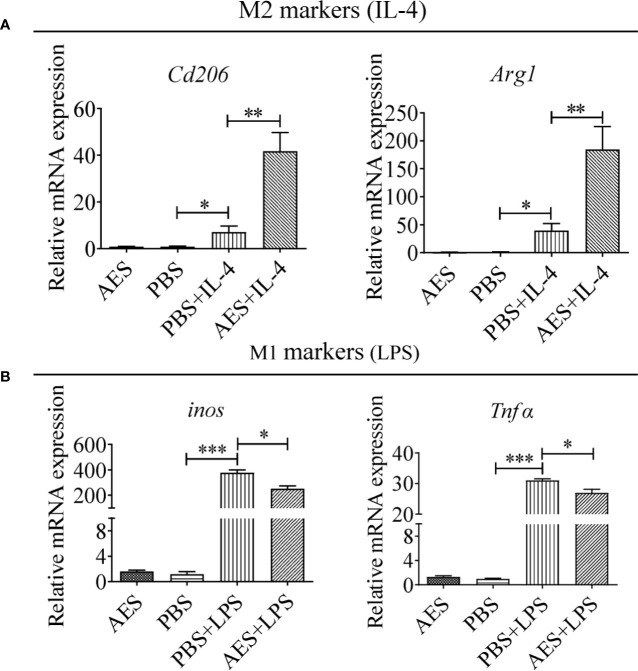
*T. spiralis* AES enhanced IL-4-induced RAW264.7 macrophage M2 polarization and decreased LPS-induced M1 polarization. RAW264.7 cells were pretreated with *T. spiralis* AES prior to stimulation with IL-4 or LPS. **(A)** The mRNA levels of M2-associated *Cd206* and *Arg1* in IL-4 stimulated RAW264.7 were analyzed by qRT-PCR. **(B)** The mRNA levels of M1-associated *inos* and *Tnfα* in LPS-stimulated RAW264.7 were analyzed by qRT-PCR. The relative expression level was compared with PBS control group as baseline. Experiments were repeated in triplicate. Data are expressed as means ± SEM. ^*^P < 0.05, ^**^P < 0.01, ^***^P < 0.001; ns = not significant.

To further investigate the role of AES in macrophage polarization, the monocyte/macrophage cell line RAW264.7 was used to evaluate *in vitro* stimulation of M1 and M2 phenotypes. As shown in **Figure 3A**, RAW264.7 macrophages differentiated into the M2 type to some extent following IL-4 stimulation presented by increased levels of *Cd206* and *Arg1* mRNA expression. LPS significantly induced M1 polarization of RAW264.7 macrophages expressing higher levels of *inos* and *Tnfα in vitro*. Pretreatment with AES significantly enhanced IL-4-stimulated expression of the M2 phenotype (*Cd206 and Arg1*), but only slightly reduced expression of the M1 phenotype (*inos and Tnfα*) in RAW264.7 cells as compared to those without AES treatment (**Figure 3**). Our findings further confirm that AES was able to effectively enhance IL-4-induced M2 macrophage polarization and slightly suppress LPS-induced M1 macrophage polarization *in vitro*. The *in vitro* findings (**Figure 3**) were consistent with those of AES-induced macrophage M2 polarization *in vivo* (**Figure 2**).

### *T. spiralis* AES Induced PD-1 Expression on M2 Macrophages

**Figure 4:**
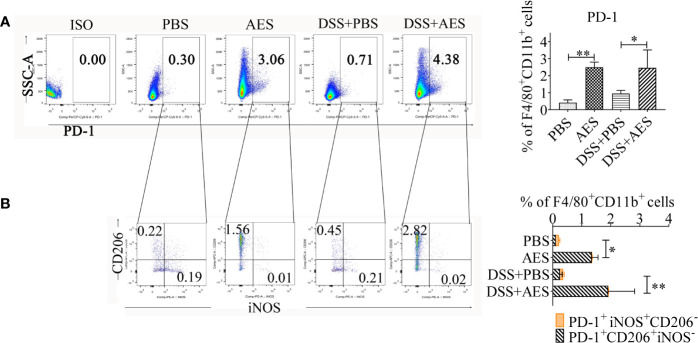
*T. spiralis* AES upregulated expression of PD-1 on M2 macrophages. Peritoneal macrophages were collected from mice of each group and expression of PD-1 on macrophages was detected by flow cytometry. **(A)** The representative side scatter area (SSC-A) dot plots of PD-1^+^ macrophages in each group. The percentages of PD-1^+^ in F4/80^+^/CD11b^+^ macrophages in each group is shown on the right panel (n=5 per group). **(B)** Representative dot plots of CD206 and iNOS (M2) expressed in PD-1^+^ macrophages (left) and the percentages of CD206^+^ or iNOS^+^ in PD-1^+^ macrophages (right) (n=5 per group). Experiments were repeated in triplicate. Data are presented as means ± SEM. ^*^P < 0.05, ^**^P < 0.01.

Recent studies suggest a critical role played by the PD-1 pathway in the modulation of macrophage polarization (24, 25, 27). Here, expression of PD-1 on peritoneal macrophages collected from AES-treated mice with DSS-induced colitis was analyzed using flow cytometry. As shown in **Figure 4**, PD-1 expression was upregulated in response to AES treatment in both normal mice and mice with DSS-induced colitis (**Figure 4A**). Further investigation revealed that AES mainly induced PD-1 expression on macrophages expressing CD206 rather than those expressing iNOS, indicating that AES mainly induced PD-1 expression on M2 but not M1 macrophages (**Figure 4B**).

### PD-1 Deficiency Offset AES-Induced M2 Macrophage Polarization

**Figure 5:**
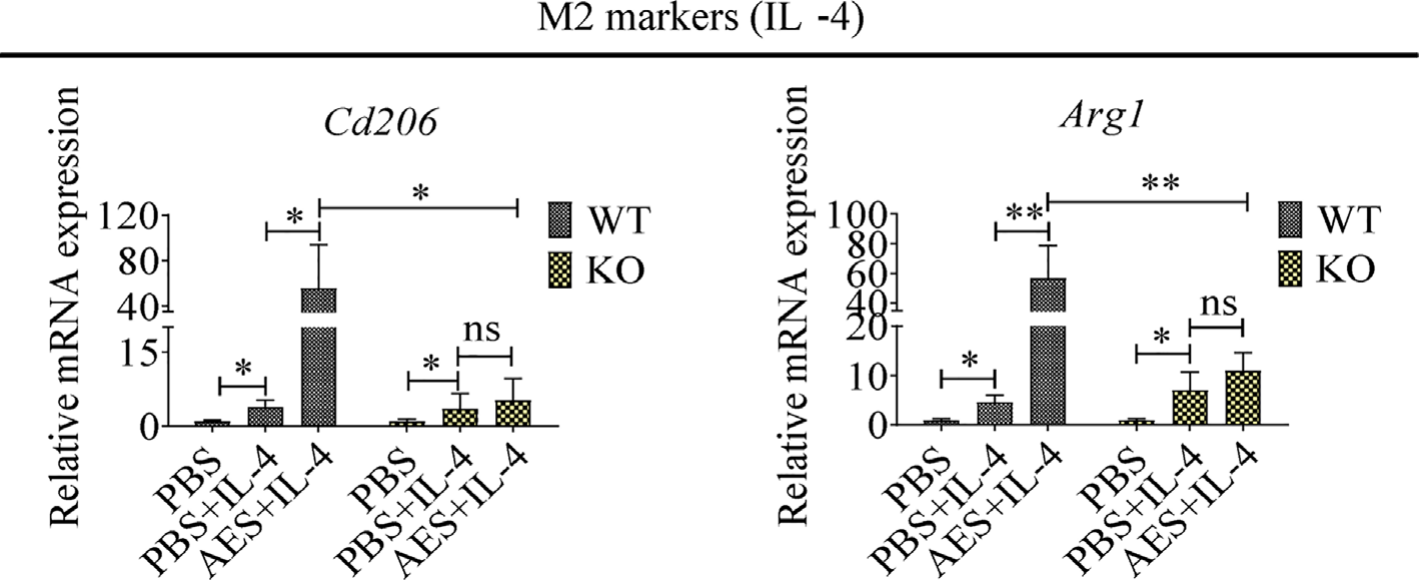
PD-1 deficiency reduced AED-induced M2 macrophage polarization, Peritoneal macrophages were isolated from wild type or PD-1 knockout mice and treated with AES. IL-4 was subsequently added to induce M2 polarization. Markers of M2 macrophages (*Cd206* and *Arg1*) were analyzed by qRT-PCR. Experiments were repeated in triplicate. ^*^P < 0.05, ^**^P < 0.01; ns = not significant.

To further investigate the role of PD-1 in AES-induced M2 macrophage polarization, peritoneal macrophages were collected from wild-type C57BL/6 mice (WT) and PD-1 KO mice (KO). Under IL-4 stimulation, the M2 markers *Cd206* and *Arg1* were found to be highly upregulated on peritoneal macrophages from WT mice when coincubated with AES. Stimulating effects of IL-4 itself on *Cd206* and *Arg1* expression were also noted. However, AES-induced M2 polarization was not observed in peritoneal macrophages from PD-1 KO mice under similar conditions (no significantly increased *Cd206* or *Arg1* expression) (**Figure 5**), further indicating that PD-1 is critical to M2 macrophage Polarization.

### PD-1 Deficiency Reduced the Therapeutic Effect of AES on DSS-Induced Colitis

**Figure 6:**
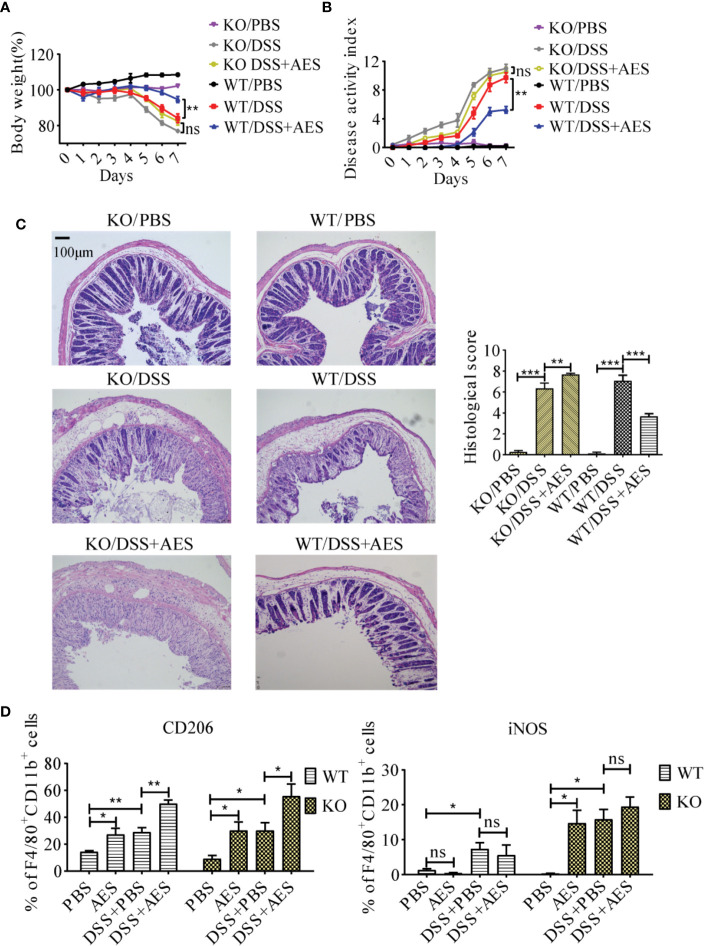
PD-1 deficiency offset the protective effects of AES on DSS-induced colitis. The WT C57BL/6 mice (WT) and PD-1 knockout mice (KO) were divided into 3 groups with 6 mice each, treated with DSS, DSS+AES, and PBS, respectively. **(A)** Quantification of weight loss in different groups (n=6). **(B)** Clinical disease activity index (DAI) was evaluated throughout the experimental period (n=6). **(C)** Microscopic histological damage (left) and score (right) in different treatment groups (n=5). **(D)** PD-1 deficiency enhanced both M1 (iNOS) and M2 (CD206) expression in PD-1 knockout mice with DSS-induced colitis treated with AES as compared to untreated mice (n = 6). Experiments were repeated in triplicate. All data are expressed as means ± SEM. *P< 0.05, **P< 0.01, ***P< 0.001.

To understand the role of PD-1 in the therapeutic effect of AES on inflammatory colitis in mice, colitis was induced in PD-1 KO mice and then treated with AES. The therapeutic results of *T. spiralis* AES was observed in WT mice with DSS-induced colitis, including reduced weight loss and total disease activity index; however, the clinical signs of colitis were not improved in PD-1 KO mice upon treatment with *T. spiralis*-AES (**Figures 6A, B**). The histological inflammation and pathology of colitis in PD-1 KO mice was even worse upon treatment with *Ts*-AES than those without AES treatment as evidenced by widely disrupted tissue architecture, the disappearance of intestinal crypts and goblet cells, marked mucosal hypertrophy and edema (**Figure 6C**). These data indicate that PD-1 plays a critical role in ameliorative effect of AES on DSS-induced colitis. Flow cytometry analysis on the peritoneal macrophages collected from experimental mice showed that CD206 (M2) was significantly upregulated, and iNOS (M1) was not increased on macrophages collected from WT mice with DSS-induced colitis and AES treatment. However, treatment with both AES and DSS significantly increased the percentage of M1-type (iNOS) macrophage in PD-1 KO mice. The iNOS percentage was increased in PD-1 KO mice with colitis treated with AES than those without AES treatment; however, the difference was not significant. The results indicate that the deficiency of PD-1 stimulated M1 macrophages. Nevertheless, the M2 marker (CD206) was also increased in macrophages collected from PD-1 KO mice upon treatment of AES (**Figure 6D**).

## Discussion

Helminth exposure tends to induce both innate and adaptive immune regulatory circuitry. Increasing evidence has suggested that helminths and their secreted products have therapeutic potential in the control or prevention of immune hypersensitivity-mediated allergic and inflammatory diseases (4, 34). The regulatory or tolerogenic phenotype of immune cells, including DCs, B cells, T cells, and macrophages, can be evoked by parasite-derived products (36).

Macrophages, especially the M1, are involved in the inflammatory process. Depletion of macrophages using CloA significantly reduced DSS-induced colitis in this study, indicating macrophages are the effector in the inflammatory colitis. Regulation of macrophage activity and function is essential for balancing tissue immune homeostasis as well as driving or resolving inflammation in most pathologic processes. Helminth protein was reported to ameliorate autoimmune diseases by enhancing M2 polarization (18) and passive transfer of *in vitro* differentiated M2 macrophages, significantly reducing the severity of DNBS-induced colitis in mice (16). However, the underlying mechanism of macrophage-based helminth therapies is yet to be understood. In this study, we investigated the effects of *T. spiralis* AES on macrophage polarization in mice with DSS-induced colitis. The proportion of M1 macrophages was found to be significantly increased in mice with DSS-induced colitis as compared with normal control mice. Given the proinflammatory nature of M1-type macrophages, these results suggest that M1-type macrophages play a major role in the pathogenesis of DSS-induced inflammation. However, AES treatment shifted M1 to M2 polarization in mice with DSS-induced colitis (**Figure 2**), indicating the capacity of AES to modulate macrophage polarization in the setting of this condition. M2 macrophages have been reported to be important for tissue repair and possess therapeutic potential against autoimmune diseases (37). We further used the mouse macrophage cell line RAW264.7 to investigate immune modulation of *T. spiralis* AES on macrophage polarization *in vitro*. Pretreatment of macrophages with *T. spiralis* AES significantly induced M2 polarization with higher expression of *Cd206* and *Arg1* on the surface of macrophages after IL-4 stimulation. Meanwhile, the expression of *inos* and *Tnfα*, the biomarkers of M1 macrophages, in the setting of LPS induction was suppressed (**Figure 3**). These findings are consistent with prior research in which *T. spiralis* secretory proteins were found to suppress *inos* expression in LPS-induced bone marrow-derived and J774A.1 macrophages (16, 38).

PD-1 plays a critical role in maintaining host immune homeostasis during chronic infection (20). The costimulatory pathway consists of PD-1 and its ligands, PD-L1 and PD-L2, delivering inhibitory signals that regulate balance among immune cells to prevent overactive inflammation (39). Our previous study reported increased expression of PD-1 on CD4^+^T cells of mice infected with *T. spiralis* (28). Other studies have demonstrated that PD-1 is also involved in modulating innate immune cell function (32, 40, 41). In addition, PD-1 deficiency has been reported to enhance M1 polarization in zymosan-induced inflammation (24, 25). More recent studies have also detailed the complicated relationship between macrophages and the PD-1/PD-L1 pathway (42). Given the known impact of the PD-L/PD-1 axis on macrophage differentiation, we investigated the role of PD-1 in *T. spiralis* AES-induced M2 polarization. Here, we observed significant upregulation of PD-1 expression in macrophages of mice treated with *T. spiralis* AES. Such increased macrophage expression of PD-1 positively correlated with the M2 phenotype (**Figure 4**). In addition, we identified that M2 polarization induced by *T. spiralis* AES *in vitro* upon stimulation of IL-4 was effectively diminished in macrophages collected from PD-1 KO mice (**Figure 5**). Recent tumor cell research also revealed that PD-1^+^ tumor-associated macrophages expressed an M2-like surface profile, and that M2 macrophages expressed significantly more PD-1 than did M1 macrophages (26). Other studies also suggested that macrophages highly expressed PD-1 provided negative feedback, which was related to downregulated expression of iNOS and proinflammatory cytokines (25, 43). Our findings reveal that *T. spiralis* AES skewed the macrophage population toward the M2 phenotype, likely *via* activation of the PD-1 pathway. Our results underscore the importance of PD-1 in modulating the balance of M1/M2 polarization upon *T. spiralis* AES exposure, suggesting one likely molecular mechanism behind helminth-induced immunomodulation and the therapeutic efficacy of helminth-derived proteins on inflammatory immune diseases. Our *in vitro* experiments also confirmed that PD-1 deficiency significantly reduced AES-enhanced M2 polarization of peritoneal macrophages upon IL-4 stimulation. Deficiency of PD-1, however, did not significantly reduce AES-stimulated expression of CD206 (M2) *in vivo* even though expression of iNOS (M1) was significantly increased on macrophages of PD-1 KO mice (**Figure 6D**). It is likely that KO of PD-1 in mice not only influences M1/M2 differentiation but also other immune response pathways that may affect macrophage expression of CD206.

It has been well established that M1 and M2 macrophages play opposing roles in DSS-induced colitis. Although M1 macrophages contribute to the pathogenesis of DSS-induced colitis *via* secretion of proinflammatory cytokines that cause tissue damage, M2 macrophages are vital in the attenuation of DSS-induced colitis, primarily by expressing anti-inflammatory cytokines (44). Adoptive transfer of *Trichinella spiralis*-activated macrophages was reported to ameliorate DSS-induced colitis in murine models (16). Given that PD-1 is critical for enhancing AES-induced M2 polarization, we investigated the role of PD-1 in the protective effects exerted by AES on DSS-induced colitis. In this study, we found that treatment with *T. spiralis* AES significantly reduced DSS-induced colitis in normal mice, but not in PD-1 knockout mice. Interestingly, treatment with AES even increased the histological score of DSS-induced colitis in PD-1 deficient mice (**Figure 6**). We further identified that M2 polarization is correlated with AES-induced mitigation of colitis, which is associated with upregulation of PD-1 expression in macrophages. Therefore, we concluded that the therapeutic effects of *T. spiralis* AES on inflammatory colitis was through driving PD-1-mediated M2 macrophage polarization. However, the signal pathway involved in the stimulation of PD-1 expression and the associated M2 polarization induced by *T. spiralis* AES is still unknown. In cancer studies, it has been identified that PD-1 expression by tumor-associated macrophages was associated with protumor M2 polarization (26). Inhibition of the PD-1 pathway increased the M1 polarization of macrophages through reducing the phosphorylation of signal transducer and activator of STAT1 (25) and increasing STAT6 phosphorylation (24). Inhibition of PD-L1 on tumor-associated macrophages increased the expression of multiple macrophage inflammatory pathways (45). It is needed to study the potential pathway involved in the activation of PD-1 and M2 polarization upon the treatment of *T. spiralis* AES and their association with reduced colitis. We postulate that PD-1-mediated M2 polarization upon *T. spiralis* AES treatment promotes an inflammation-suppressed environment, which is beneficial to the amelioration of DSS-induced colitis.

In this study, we have confirmed the therapeutic effect of *T. spiralis* AES on DSS-induced inflammatory colitis associated with PD-1 mediated M2 macrophage polarization. However, the AES are a complex pool of various molecules secreted or excreted by the worms, which are not safe as therapeutic reagents and also not feasible for large-scale production. It is necessary to identify the specific molecules in the ES products involved in immunomodulation (46). LC-MS/MS identified more than 280 protein components in *T. spiralis* AES with 4 proteins having potential regulatory functions that include cysteine protease inhibitor, serine protease, 53 kDa excretory/secretory antigen, and glutathione-S-transferase (47–49). Some of the proteins secreted by the *T. spiralis* adult worm have revealed regulatory functions to the host immune system. The *T. spiralis* secreted serine protease alleviated the severity of TNBS-induced colitis by balancing the CD4^+^ T cell immune response (50). Paramyosin from *T. spirialis* (*Ts*-Pmy) plays an important role in modulating the host immune system by inducing regulatory T cells (51). The identification of the immunomodulatory molecules in the ES products make it possible to manufacture the products as reagents for therapeutic targets for inflammatory and immune diseases, which are greatly needed, and also for vaccine targets to prevent infection of the parasite. This study is under investigation in our lab.

To sum up, our findings underline the importance of macrophage in pathogenesis of colitis. *T. spiralis* AES attenuated DSS induced colitis in mice *via* M2-type macrophage, and the polarization of M2-type macrophages is partly dependent on the PD-1 pathway. Our findings advance understanding of the protective mechanisms of *T. spiralis* AES on colitis and promote the development of *T. spiralis* AES and their derived protein(s) as potential biological agents to treat inflammatory and autoimmune diseases. Further studies are needed to explore the mechanisms and pathways of helminth-induced PD-1-mediated immunomodulation and identify the specific protein(s) in *T. spiralis* AES that play regulatory function on host immune system.

## Data Availability Statement

All datasets presented in this study are included in the article/supplementary material.

## Ethics Statement

The animal study was reviewed and approved by: The protocols for animal experiments were approved by Capital Medical University Animal Care and Use Committee on the Ethics of Animal Experiments (Permission No. AEEI-2017-133.).

## Author Contributions

XZ designed the project and coordinated experimental work. ZW, CH, QZ, XS, and JH carried out experimental work. ZW, YC, BZ, and XZ wrote the manuscript with valuable input from all other authors. All authors contributed to the article and approved the submitted version.

## Funding

This study was funded by the National Natural Science Foundation of China (81672042, 81572016).

## Conflict of Interest

The authors declare that the research was conducted in the absence of any commercial or financial relationships that could be construed as a potential conflict of interest.

## Abbreviations

IBD, inflammatory bowel disease; CloA, liposomal clodronate, Clophosome -A; PD-1, programmed death 1; WT, wild type; KO, knockout; DAI, disease activity index; MPO, myeloperoxidase; DSS, dextran sulfate sodium; AES, excretory/secretory products from *Trichinella spiralis* adult worms; FVD, fixable viability dye.
